# Atypical tibial fracture in breast cancer patient with bone metastasis receiving denosumab therapy: a case report and review of the literature

**DOI:** 10.1186/s13256-023-03999-7

**Published:** 2023-06-21

**Authors:** Yohei Yanagisawa, Hidefumi Suzuki, Hisanori Gamada, Masashi Yamazaki

**Affiliations:** 1 Department of Emergency and Critical Care Medicine, University of Tsukuba Hospital, 2-1-1 Amakubo, Tsukuba, Ibaraki 305-8576 Japan; 2 Department of Orthopaedic Surgery, University of Tsukuba, 1-1-1 Tennodai, Tsukuba, Ibaraki 305-8575 Japan

**Keywords:** Atypical fracture, Denosumab, Breast cancer, Skeletal-related events, Metastatic bone disease, Atypical tibia fracture

## Abstract

**Background:**

Denosumab therapy is often used to reduce skeletal-related events in metastatic bone disease. On the other hand, there have been some instances of atypical femoral fracture in patients with metastatic bone disease treated with denosumab. In this case report, we describe a patient with metastatic bone disease due to breast cancer who had been using denosumab for 4 years to prevent skeletal-related events and suffered an atypical tibial fracture.

**Case presentation:**

We report here the case of an 82-year-old Japanese woman who had received yearly intravenous denosumab for 4 years and presented with a fracture fulfilling the criteria for an atypical fracture, except for being located at the tibial diaphysis. She was found to have stage 4 breast cancer with multiple bone metastases 4 years prior. She had difficulty walking due to her tibial pain and underwent surgical treatment. Four months after surgery, the tibial fracture site exhibited bone fusion.

**Conclusion:**

In patients with long-term use of denosumab to prevent skeletal-related events in metastatic bone disease, it is important to be aware of shin and thigh pain and to examine for signs of atypical tibial fractures to pay attention to atypical femoral fractures.

## Background

Antiresorptive drugs are effective in patients with breast cancer-associated bone metastases and prevent the occurrence of skeletal-related events (SREs). These drugs include zoledronic acid, pamidronate, and denosumab [1–4]. Denosumab therapy is often used to reduce SREs in metastatic bone disease (MBD) [5]. On the other hand, in the literature where the effects of denosumab have been reported, there have been some instances of atypical femoral fracture (AFF) in patients with MBD treated with denosumab [5–7]. In this case report, we describe a patient with MBD due to breast cancer who had been using denosumab for 4 years to prevent SREs and suffered an atypical tibial fracture (ATF). We report this case because it is a rare case of an ATF, probably the first known instance, which may have been induced by denosumab.

## Case presentation

We report here the case of an 82-year-old Japanese woman who had received yearly intravenous denosumab for 4 years and presented with a fracture fulfilling the criteria for an atypical fracture, except for being located at the tibial diaphysis. There was no history of bisphosphonate or oral glucocorticoid use. She was found to have stage 4 breast cancer with multiple bone metastases 4 years prior. Since then, she has been treated with an aromatase inhibitor and denosumab.

**Fig. 1:**
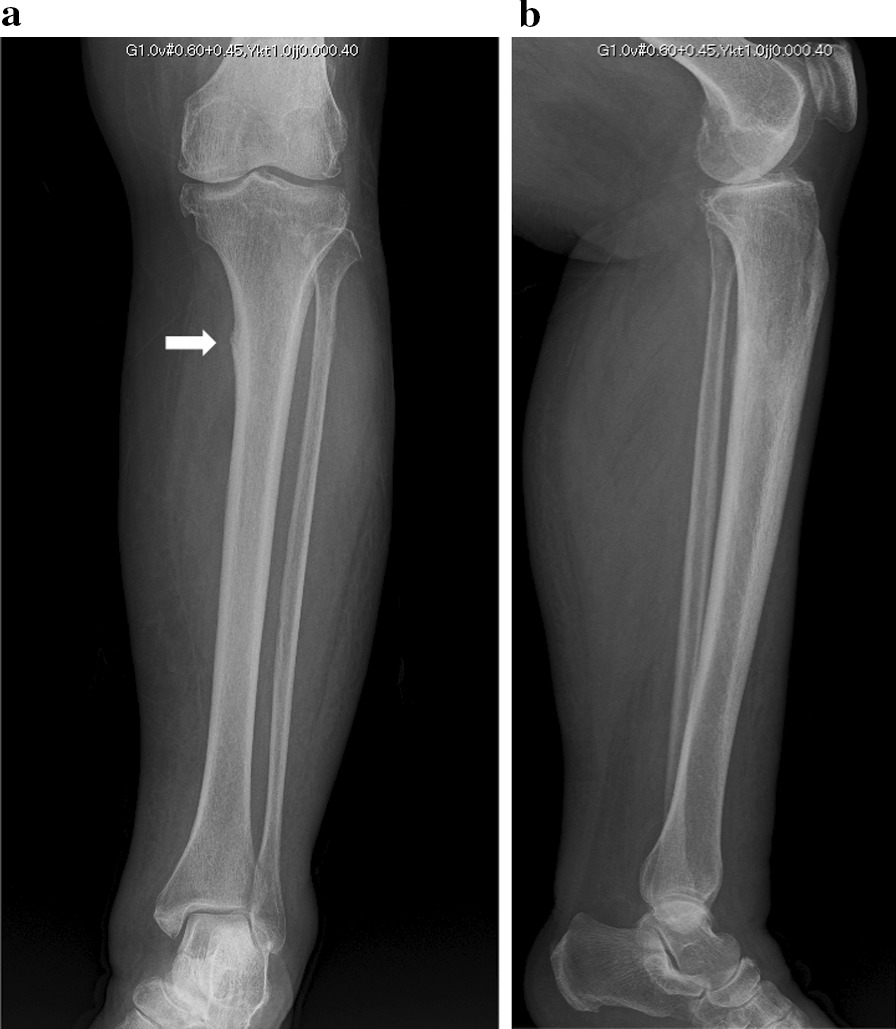
X-ray images at the initial visit for her shin pain. The “beaking sign” (white arrow) occurred due to focal endosteal thickening of the medial cortex in the proximal third of the tibia

The patient had no obvious history of trauma. One day, while walking, pain appeared in her left shin. A few days after the pain occurred, she visited an outpatient orthopedic clinic. X-ray images from the initial visit are shown below (Fig. 1a and b).

**Fig. 2:**
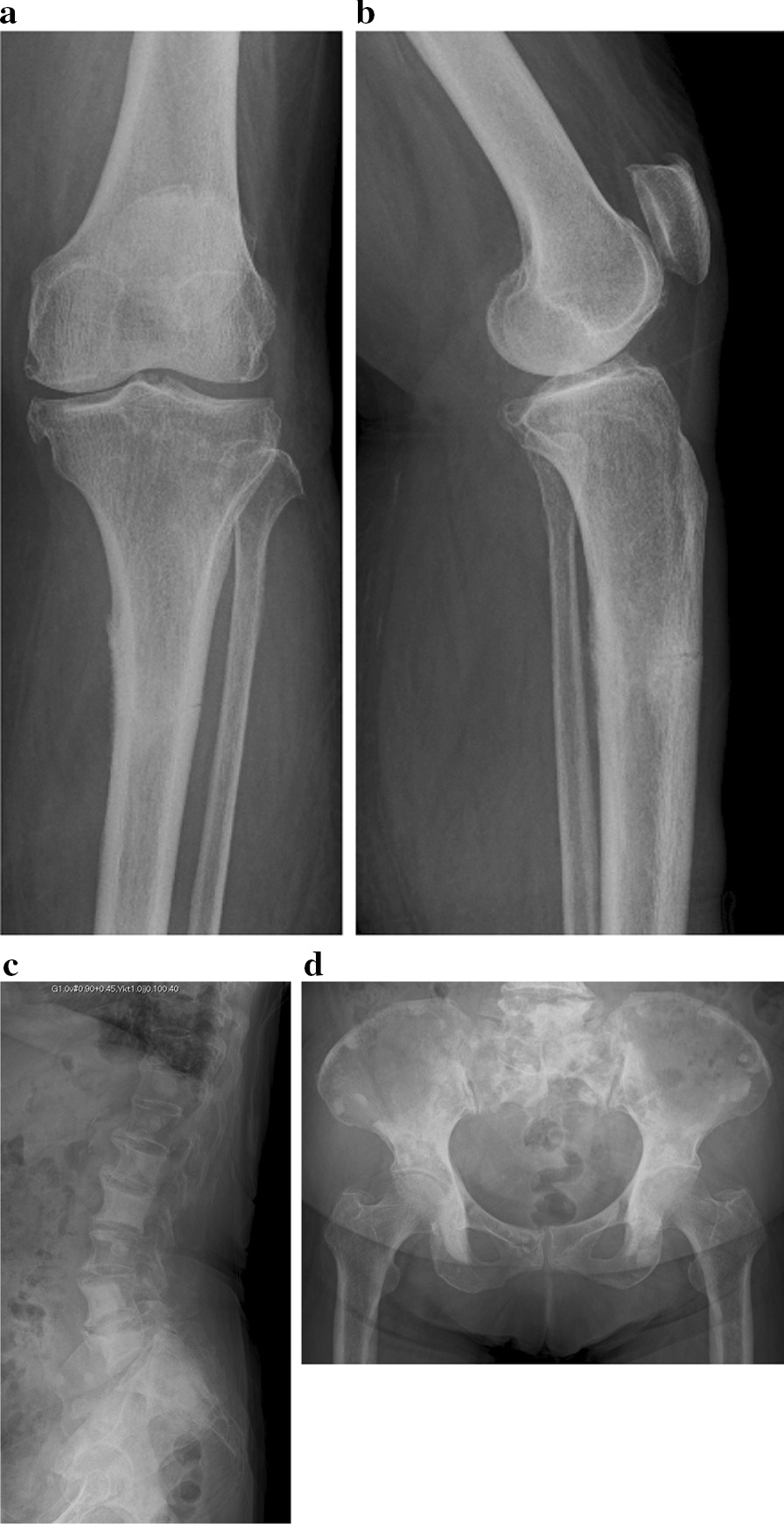
X-ray image demonstrated a non-displaced transverse fracture of the proximal third of the left tibial diaphysis. **a** Anteroposterior (AP) view of the left tibia. **b** Lateral view of the left tibia. **c** Lateral view of the lumber spine. There were multiple metastases of lumber bodies; **d** anteroposterior X-ray of pelvis. There were also multiple bone metastases

Because her fracture was without any accompanying dislocation, she was followed up at home with full weight bearing on her left leg. Her pain persisted for a month after the initial visit, and she had difficulty walking because of the shin pain. She returned to the orthopedic outpatient clinic, and X-ray images demonstrated a non-displaced transverse fracture of the proximal third of the left tibial diaphysis (Fig. 2a and b).

She had multiple bone metastases to several spinal and pelvic bones 4 years prior, due to breast cancer. (Fig. 2c and d).

The tibial fracture was also considered a differential for a pathological fracture due to a metastatic bone tumor. Therefore, the patient underwent additional imaging examination, including CT and MRI scans. The CT and MRI images did not show any positive findings to suggest a metastatic bone tumor, but they could not rule out the possibility either. At the time of the fracture, the serum biochemical parameters of bone metabolism and cancer markers were 25-hydroxyvitamin D 32 ng/mL, total calcium 10.3 mg/dL, tartrate-resistant acid phosphatase 5b (TRACP-5b) 192 mU/dL, carcinoembryonic antigen (CEA) 66.3 ng/mL, and cancer antigen 15-3 (CA15-3) 109.7 U/mL.

Her tibial fracture pattern fulfilled all the major criteria (except for the location) and several minor criteria of an atypical fracture. We suspected from these findings that her tibial fracture was atypical.

**Fig. 3:**
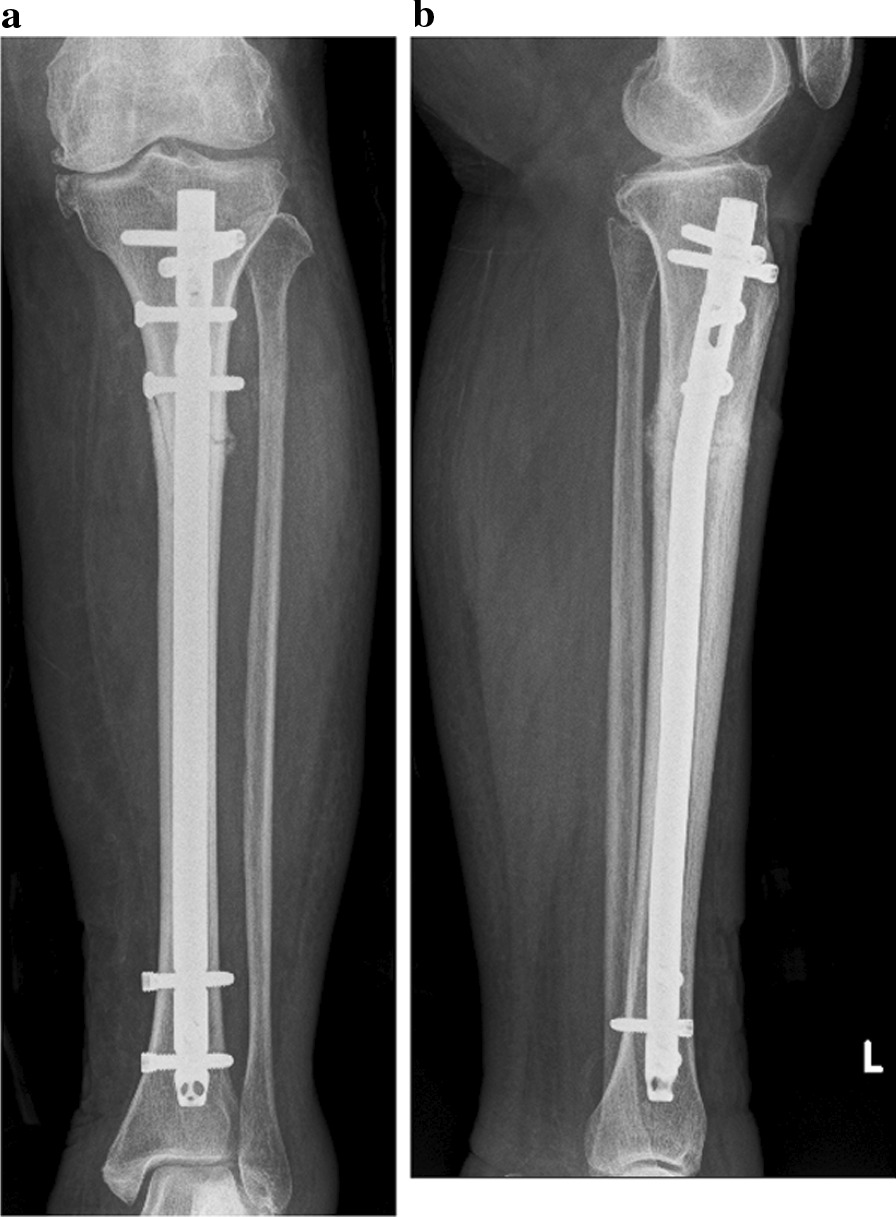
Two-directional X-ray image of her lower leg just after surgery. The intramedullary nail used was the Tibial Nail Advanced (φ10.0 mm, length 270 mm, DePuy Synthes, Paoli, PA)

**Fig. 4:**
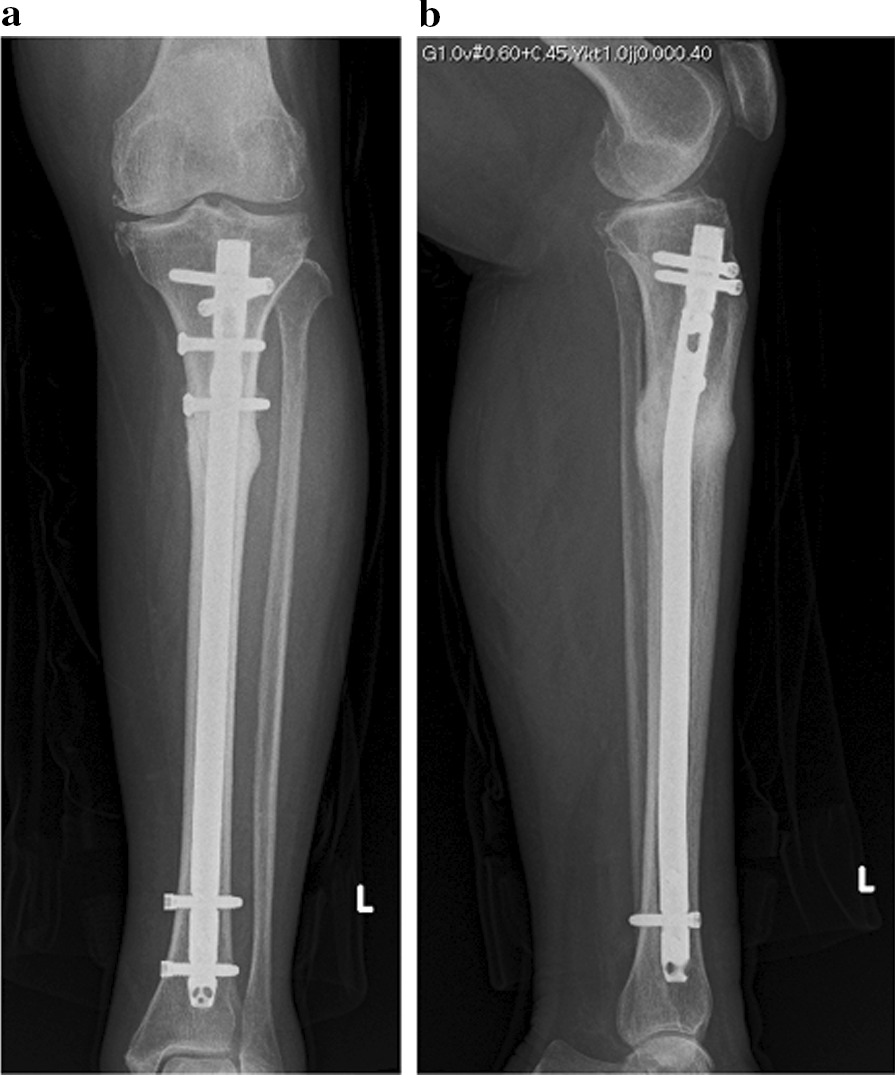
Two-directional X-ray image of her lower leg 4 months after surgery. The fracture was united

She had difficulty walking due to pain and underwent surgical treatment. Treatment consisted of an intramedullary nail that immediately resolved the pain (Fig. 3a and b). The cancellous bone within the medullary cavity of the fracture was harvested during intramedullary reaming. The skin near the medial fracture site was also incised, and external periosteum and bone tissue around the fracture site were collected. Pathological examinations were performed on both bone tissue samples. Both samples had no neoplastic changes that could be considered breast cancer-associated bone metastases. Therefore, a pathological fracture due to bone metastases from breast cancer was ruled out, and we diagnosed her tibial fracture as an ATF. Four months after surgery, the fracture site exhibited bone fusion (Fig. 4a and b).

Upon literature review, we discovered that only three previously reported cases of atypical tibia fracture with denosumab had been published. We searched the PubMed database for papers related to atypical tibia fractures and discovered 97 references. After checking all of the titles and abstracts, we found three reports of atypical tibia fractures related to denosumab use [8–10]. The results are shown in Table 1.

**Table 1:** .

Author (Year)	Age (years)	Sex	Diagnosis	Antiresorptive agent (duration)	Fracture site	Management
Sian *et al.* [8] (2015)	60	M	Giant cell tumor	Denosumab (120 mg/month, 4 years)	Bilateral tibial shaft	Conservative treatment
Schimpf *et al.* [9] (2018)	76	F	Rheumatoid arthritis, glucocorticoid-induced osteoporosis	Risedronate (5 years), Strontium ranelate (2 years), Zoledronate (4 years), Denosumab (60 mg/6-monthly, 2 years)	Bilateral distal tibial metaphyseal fractures	Plate fixations Intramedullary nail fixation
Juan *et al.* [10] (2019)	35	F	Juvenile idiopathic arthritis	Alendronate, teriparatide, denosumab (2 years, dosage not mentioned)	Bilateral tibial diaphyseal fracture	Intramedullary nail fixation

In one case, the patient had a giant cell tumor of the femur and was administered 120 mg denosumab once a month, the same dose as in this case. All three reports described bilateral tibial lesions. The patient in this study had no pain in the right tibia and no cortical thickness (beaking sign), as was observed in the X-ray images of her left tibia, in the right tibia. Informed consent for the acquisition of data to present in this case report was obtained during the hospitalization, and signed by the patient herself.

## Discussion and conclusions

A retrospective analysis of patients who were administered denosumab for MBD revealed the incidence of AFF to be 0.4%–1.8%. AFF is a known adverse event associated with the use of denosumab [6, 7]. Osteoporosis is treated with 60 mg of denosumab every 6 months, whereas bone metastases are treated with 120 mg once a month. The incidence of AFF with denosumab usage at doses used to treat osteoporosis is very rare, with Henry *et al.* reporting only one out of 2626 cases [11]. Takahashi *et al.* reported that long-term administration of denosumab (at a dose of 120 mg) is a risk factor for AFF in patients with MBD, as is zoledronic acid [7].

It is unclear at this time whether the risk of developing AFF also pertains to the risk of developing ATF. However, as mentioned below, the patient had a history of high-dose, long-term denosumab and Herceptin use, which have been considered risk factors for the development of AFF in patients with MBD in the previous literature [6, 7]. In this case, the drug was used to prevent SREs and was injected at a dose of 120 mg once a month. When used in the treatment of osteoporosis, it is given once every 6 months, but 120 mg is injected monthly to prevent SREs in patients with MBD. The duration of injections has also been reported to be a risk factor for AFF in patients with MBD. In particular, it is noted that administering denosumab for more than 3.5 years poses a risk [7]. In this case, the patient had received denosumab for an extended period of 4 years. Long-term denosumab injections and zoledronic acid administration prior to denosumab have also been identified as a risk factor for AFF in patients with MBD [7]. There was no history of prior zoledronic acid use in this patient. In addition, the risk of AFF in patients with MBD during high-dose denosumab administration is increased with the concomitant use of aromatase inhibitors, in addition to long-term denosumab and prior zoledronic acid usage [6]. The patient continued to use Herceptin in combination with denosumab during the 4-year period, which could have been a risk factor for fractures.

If a low bone metabolic turnover caused atypical femoral [12], ulnar [13, 14], and tibial fractures, as described in previous reports [10], then it is possible that it also contributed to the atypical fractures in this patient. On admission, the TRACP-5b level was measured to be 192 mU/dL, suggesting that osteocyte activity was suppressed.

**Fig. 5:**
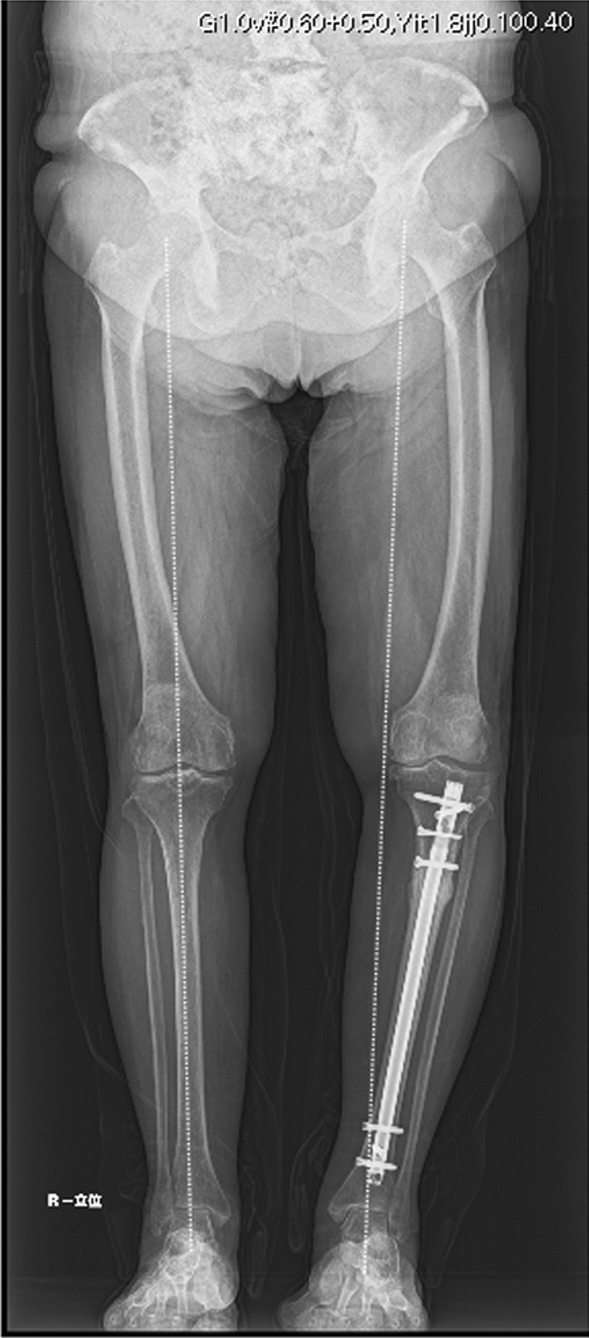
X-ray image of the lower limbs of the patient in the standing position, and the axis of lower extremity loading. The dotted lines indicate the bilateral Mikulicz’s lines of the lower extremity loading axis

Other reports suggest that lower extremity loading stress associated with the lower extremity loading axis influences the site of AFF [15]. The relationship between the frontal X-ray image of the standing position and the axis of lower extremity loading is shown in this case (Fig. 5). In this patient, the Mikulicz’s line of the lower extremity loading axis passed through the medial aspect of the knee joint, which was expected because of the O-shaped leg deformity and the internal rebound force on the proximal tibial diaphysis.

In conclusion, the patient had received denosumab at a high dosage for a long period of time (4 years) with no prior exposure to zoledronic acid. On the other hand, there was a history of aromatase inhibitor use while on denosumab. These circumstances suggest that this patient had multiple risk factors for atypical fractures and that her tibial fracture might be atypical. In patients with long-term use of denosumab to prevent SREs in MBD, it is important to be aware of shin pain to examine for signs of ATFs, as we do to be aware of thigh pain for AFFs.

## Data Availability

All the required information is available in the manuscript.

## Abbreviations

AFF: Atypical femoral fracture
ATF: Atypical tibial fracture
MBD: Metastatic bone disease
SRE: Skeletal-related events
TRAP-5b: Tartrate-resistant acid phosphatase 5b
